# The effect of an osteoarthritis adaptation program on biological, psychological, and social integrity variables and quality of life in older women with osteoarthritis in Korea: a quasi-experimental study

**DOI:** 10.7586/jkbns.25.036

**Published:** 2025-08-22

**Authors:** Hye Sim Seo, Young Eun, Mi Yang Jeon

**Affiliations:** 1 Department of Nursing, Kwangju Women's University, Kwangju, Korea; 2 College of Nursing, Sustainable Health Research Institute, Gyeongsang National University, Jinju, Korea

**Keywords:** Adaptation, physiological, Adaptation, psychological, Aged, Osteoarthritis

## Abstract

**Purpose:**

This study aimed to determine whether an osteoarthritis adaptation program increased biological, psychological, and social integrity variables and quality of life in older women with osteoarthritis in Korea.

**Methods:**

This study employed a non-equivalent control group pretest–posttest design, allocating 51 older women with osteoarthritis to an experimental group (26 participants) and a control group (25 participants). The program was conducted thrice weekly for 12 weeks, with each session lasting 60 to 90 minutes. It combined exercises (flexibility, muscle strength, and aerobic) and education (pain control, nutrition management, and compliance).

**Results:**

Pain differed significantly between the groups (F = 12.10, *p* < .001), and the time-group interaction was also significant (F = 23.65, *p* < .001). Difficulty in daily living did not show significant differences between the groups (F = 3.88, *p* = .055), although the time-group interaction was significant (F = 39.57, *p* < .001). Depression showed a significant difference between the groups (F = 7.24, *p* = .010), as did the time-group interaction (F = 9.17, *p* = .002). Social support also differed significantly between the groups (F = 46.07, *p* < .001), and the time-group interaction was significant (F = 45.22, *p* < .001). Quality of life showed significant differences between the groups (F = 29.04, *p* < .001), and the time-group interaction was also significant for this variable (F = 64.32, *p* < .001).

**Conclusion:**

The osteoarthritis adaptation program effectively improved biological, psychological, and social integrity variables in older women with osteoarthritis. Therefore, this program offers considerable benefits to older women with chronic osteoarthritis, emphasizing its value for individuals with osteoarthritis living in the community.

## INTRODUCTION

In 2025, the proportion of the elderly population (65 years and above) in Korea reached 20.3%, becoming a super-aged society [1]. The estimated prevalence of osteoarthritis (OA) was 25.0% (10.3% for men and 35.9% for women) among elderly Koreans aged 65 years and older [2]. Thus, care for older adults with OA is necessary in an aging society, and joint care for older women is even more important.

OA is a condition in which cartilage is lost with age and the joint is deformed, causing local degenerative changes. Without cartilage, the joint ends grow abnormally and become irregular, causing pain from friction between the bones [3]. OA typically presents as joint pain that is exacerbated by use and alleviated with rest. There is a relatively brief, self-limiting morning stiffness and an absence of constitutional symptoms. Although pharmacological and non-pharmacological interventions are generally effective in relieving pain and improving physical function, they do not fundamentally reverse the pathological and radiographic processes of OA. As the severity of the disease increases, the magnitude of pain and functional impairment intensifies [4]. Therefore, to alleviate pain and improve the physical functions of older women with OA so that they can perform daily-life activities, OA must be understood and adapted to as an illness. Therefore, nursing interventions that promote adaptation of patients to OA are necessary.

In Roy's adaptation model, adaptation is defined as a process of responding positively to environmental changes [5,6]. Roy's adaptation model classified various stressors into focal stimuli, related stimuli, and residual stimuli. Focal stimuli are internal or external stimuli that immediately confront people, and are immediately encountered events or situational changes that have the greatest influence on human behavior among various stimuli. Contextual stimuli are other identifiable factors that contribute to the effect of focal stimuli, and are all environmental factors that exist inside or outside the human system. Residual stimuli are factors whose effects are unclear in the current situation, and whose effects on the current situation are uncertain. Adaptation is expressed as physiological and physical, self-concept, role function, and interdependence through coping mechanisms consisting of regulatory and cognitive mechanisms. The physiological and physical mode is the physical response related to interacting with the environment to meet the basic needs of oxygenation, nutrition, excretion, activity, rest, and protection. The self-concept mode focuses on the mental and spiritual aspects of a person, and is the perception of oneself or the subjective idea of one's body. The role functioning mode focuses on the role that the individual occupies in society. It is interacting with other people in the environment or performing the role skillfully with appropriate behavior. The interdependence mode focuses on interdependence related to giving and receiving love, respect, and value, and is related to the support system with others. This adaptive response enhanced human integration. Moreover, to adapt to life, patients with OA must successfully cope with the pain and changes caused by OA to maintain and improve their quality of life [6].

In Roy's adaptation model [5], adaptation is a process of responding positively to environmental changes. These adaptive responses are expressed as physiological-physical, self-concept, role function, and interdependent adaptation modes. The adaptation of older women with OA is a process of improving quality of life through physiological, psychological, and social integration. This is achieved by affecting physiological, physical, self-concept, role function, and interdependent adaptation modes through the coping mechanisms of cognition and regulation concerning changes in life due to pain. Nevertheless, most joint management programs [7-10] for patients with OA reported that exercise and education programs increase pain, physical function, and activities of daily living, or reduce exercise self-efficacy [11] or depression [12]. However, there has been a significant lack of research attempting to change the adaptation mode by strengthening the coping mechanisms of patients with OA and thereby improving quality of life.

Therefore, we aimed to test the effects of the OA adaptation program based on Roy's adaptation model, which improves cognitive and regulatory functions, to assist with the adaptation of older women with OA.

## METHODS

### 1. Study design

This quasi-experimental study used a non-equivalent control group pretest–posttest design to test the effects of a program on older women with OA.

### 2. Participants

The participants were older women with OA in Jinju City who agreed to participate and met the selection criteria. The specific selection criteria were as follows: first, diagnosis of OA more than one year ago; second, older than 65 years; third, ability to perform the program without limitations in activities or cognitive disorders; fourth, ability to speak Korean; and fifth, one for whom the doctor recommended exercise.

The study's sample size was calculated using the G*Power 3.1.9.7 [13], with an effect size of .25, a significance level of .05, a power of .90, two groups, three measurements, and a correlation coefficient of .30; the required sample size was determined to be 54. The G*power program sets the default value for the between-time correlation coefficient of repeated-measures analysis of variance (ANOVA) to 0.5 [13]. However, when analyzing the difference between values measured multiple times in the same subject in repeated-measures ANOVA, the between-time correlation coefficient is high. In contrast, when analyzing the difference between values measured in different subjects, the correlation is low [14]. In this study, we set the correlation coefficient value to 0.3, which is a weak correlation, because we intended to analyze the interaction of the difference by time point as well as the difference by time point and the difference by group.

After accounting for a 20% dropout rate, 66 participants were finally enrolled. Among the 66 who met the selection criteria, 33 who wished to participate in the program were assigned to the experimental group, and 33 who could not consistently participate were assigned to the control group. Seven experimental participants (four had less than an 80% participation rate, two traveled overseas, and one was hospitalized) were excluded (dropout rate of 21.2%). In contrast, eight control participants (three discontinued contact, three visited their children living in another part of the country, and two were hospitalized) were excluded (dropout rate of 24.2%). The final sample comprised 51 participants: 26 in the experimental group and 25 in the control group.

### 3. Instruments

#### 1) Pain

Pain was measured using the numerical rating scale. A 10 cm line was marked from 0 to 10 at 1 cm intervals. Participants indicated their corresponding pain levels, which were scored (0 = no pain; 10 = unbearable pain). Higher scores indicated more severe pain.

#### 2) Perceived health status

This study was measured using the perceived health status instrument developed by Speake et al. [15]. This tool comprises three items (current health condition, health compared to friends, health condition in the past 6 months) on a 5-point scale, from “Very healthy (5 points)” to “Very unhealthy (1 point).” The scores ranged from 3 to 15 points, with higher scores indicating greater perceived health status. The Cronbach’s alpha value in this study was .82.

#### 3) Depression

This study was measured using the Geriatric Depression Scale-Short Form developed by Sheikh and Yesavage and translated by Song [16]. This tool consisted of 15 items rated on a dichotomous scale (1 = yes, 0 = no). The scores ranged from 0 to 15 points, with higher scores indicating greater depression. In Song’s study, the Cronbach’s alpha value was .94 [16], and in this study, it was .79.

#### 4) Difficulty of daily living

This study was measured using the Western Ontario and McMaster Universities Osteoarthritis Index (WOMAC Index) developed by Bellamy, Buchanan, and Goldsmith, and revised into Korean by Bae (K-WOMAC Index) [17]. Difficulty in daily living was measured using 17 items on a 5-point scale (0–4 points). Scores range from 0 to 68, with a higher score indicating greater difficulty in daily living. The Cronbach’s alpha value for the K-WOMAC Index was .96 [17], and in this study, it was .96.

#### 5) Social support

This study was measured using the elderly social support instrument developed by An [18]. This tool comprises six items on a 5-point scale, from "Strongly agree (5 points)" to "Do not agree at all (1 point)." The scores ranged from 6 to 30 points, with higher scores indicating greater social support. The Cronbach’s alpha value was .81 in An’s study [18], and in this study, it was .70.

#### 6) Quality of life

This study was measured using the Elderly Quality of Life Instrument developed by Cheoi [19]. This instrument comprises 32 items on a 5-point scale, from "Very satisfied (5 points)" to "Very unsatisfied (1 point).” Among the 32 items, we measured 28 in our study, excluding four items on marital life, financial activity, educational level, and satisfaction with sex life. The scores ranged from 28 to 140 points, with higher scores indicating greater quality of life. In Cheoi’s study, the Cronbach’s alpha value was .98 [19], and in this study, it was .93.

### 4. Intervention

#### 1) Development of the OA adaptation program

**Figure 1.**
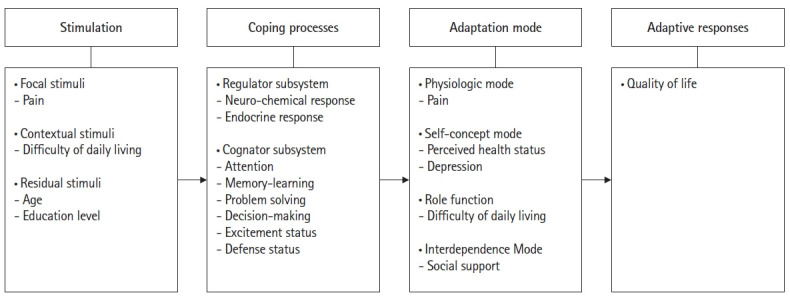
Theoretical foundation of the research.

This study reviewed previous studies [20-23] that strengthened coping mechanisms based on Roy’s adaptation model [5,6] and joint-care programs for patients with OA [7,8]. It developed a program to promote the adaptation of patients with OA. In this study, the focal stimulus of Roy’s adaptation model was chronic pain; the contextual stimulus was difficulties in daily living; and the residual stimulus was age and education level. The physiological-physical mode of Roy’s adaptation model was linked to pain; the self-concept mode was linked to perceived health status and depression; the role function mode was related to difficulties in daily living; and the interdependence mode was linked to social support. In this study, the adaptive responses of older women with OA were set as quality of life (Figure 1).

The program comprises education and exercise to promote adaptation. The program comprised 36 sessions, 60 to 90 minutes per session, held 3 times per week over 12 weeks. Education was conducted in one 15∼20 minute session per week on the topic of arthritis care. The educational topics included understanding arthritis, pain control, nutrition management, and medication. Exercise was performed in three sessions per week, comprising 40∼45 minutes (10 minutes on flexibility, 10∼15 minutes on strengthening exercises, and 20 minutes of aerobic exercise) per session.

The validity and suitability of this program’s structure and contents were confirmed by two professors in nursing schools who held instructor certificates for an OA self-help program, seven orthopedic doctors, and two physical therapists.

#### 2) Experimental group

In this study, participants in the experimental group received the OA adaptation program, which was conducted three times a week. The adaptation program was divided into two phases: Phase 1 (Weeks 1~6) and Phase 2 (Weeks 7~12).

∎ Phase 1 (Weeks 1∼6): Education sessions were held once a week, focusing on different weekly topics for 15∼20 minutes. Exercise sessions were conducted three times a week. Flexibility exercises included neck, shoulder, wrist, waist, leg, and ankle movements, repeated twice. Strength training involved both upper and lower body exercises, such as making a fist and lifting it to the chest and beside the ears, raising arms and bending them behind the shoulders, sitting and lifting the ankle, lifting the legs with force, opening legs sideways without touching the floor, and lifting legs while pressing the knee with one hand. These exercises were performed for 4 seconds and repeated twice for both right and left sides. Aerobic exercises involved rhythmic movements to the song "What about my age," with 3∼4 different movements repeated twice. Flexibility exercises included walking sideways while standing, standing on one foot, walking forward, and walking backward, consisting of 3∼4 different movements. Each exercise type—flexibility, strength training, and aerobics—was conducted twice per session.

∎ Phase 2 (Weeks 7∼12): The educational content from Phase 1 was repeated. Exercise intensity was increased as follows: During weeks 7 and 8, flexibility exercises were repeated twice, strength training three times, and aerobic exercises four times. During weeks 9 and 10, flexibility exercises were done twice, strength training four times, and aerobic exercises five times. During weeks 11 and 12, the same pattern as weeks 9 and 10 was followed, with flexibility exercises twice, strength training four times, and aerobic exercises five times per session.

#### 3) Control group

In this study, participants in the control group received only OA treatment, without any adaptation program consisting of education or exercise.

### 5. Data collection

The pretest was conducted immediately before the program. In the pretest, the researcher distributed surveys on general characteristics, level of pain, perceived health status, depression, difficulty in daily living, social support, and quality of life in both the experimental and control groups. The participants completed the questionnaires personally, or the research assistants read them, and their responses were recorded. The survey took 20∼25 minutes to complete. Using the same measurements as the pretest, the first posttest was conducted six weeks after the start of the program, and the second posttest was conducted after the termination of the 12 week program.

### 6. Statistical analysis

The data were managed using Excel, and analyses were performed using SPSS version 26.0(IBM Corp., Armonk, NY, USA). The participants' general characteristics were analyzed using frequencies, percentages, means, and standard deviations. t-test, *χ*^2^ test, and Fisher’s exact test were performed to test the homogeneity of the general characteristics of both groups. The reliability of each instrument was evaluated using Cronbach’s alpha. To test the prior homogeneity of the variables between the groups, we performed an independent t-test. To test the effect of the program, we conducted a repeated-measures ANOVA, defining illness duration as a covariate.

### 7. Ethical considerations

This study was reviewed and approved by the Institutional Review Board (IRB) of Gyeongsang National University (No. GIRB-A17-Y-0004). During the study, we adhered to the IRB guidelines. Participants were recruited offline, and only those who voluntarily agreed to participate were included in the study. After receiving a thorough explanation of the study's objectives and procedures, participants provided informed consent via a questionnaire. The questionnaire included detailed information about the study’s purpose, the protection of participants’ anonymity and personal information, and their right to refuse or withdraw from the study at any time. It was also clearly stated that they could stop the intervention during the study if they so desired. Additionally, we informed participants that all collected data would be used solely for research purposes, that responses would be anonymized, and that personal information would be stored in an encrypted file on a password-protected computer for three years before being permanently destroyed. All of the experimental and control participants received a small gift as a gesture of gratitude. One session of the program was conducted 60 minutes after the final survey to control for participants who wished to participate.

## RESULTS

### 1. Homogeneity test of the general characteristics of participants

We tested the homogeneity of the general characteristics of the study groups. Age, marital status, education level, number of children, cohabitation, whether one received joint surgery, type of insurance, financial condition, housework, and body mass index were found to be homogeneous. However, there was a statistically significant difference in the illness duration of the experimental (10.62 ± 5.65) and control groups (6.48 ± 5.35), which was not homogeneous (t = 2.68, *p* = .010) (Table 1).

In the pretest homogeneity results of the research variables, pain, difficulty in daily living, exercise self-efficacy, depression, social support, and quality of life showed no statistically significant differences between the groups; therefore, they were homogeneous (Table 2).

### 2. Effect of the OA adaptation program

The results indicated a significant difference in pain levels between the groups (F = 12.10, *p* < .001), as well as a significant time-group interaction (F = 23.65, *p* < .001). Although there were no significant differences in daily living difficulties between the groups (F = 3.88, *p* = .055), the time-group interaction was significant (F = 39.57, *p* < .001). Depression levels were significantly different between the groups (F = 7.24, *p* = .010), and there was also a significant time-group interaction (F = 9.17, *p* = .002). Social support exhibited significant differences between the groups (F = 46.07, *p* < .001), and the time-group interaction was also significant (F = 45.22, *p* < .001). Finally, quality of life demonstrated significant differences between the groups (F = 29.04, *p* < .001), as well as a significant time-group interaction (F = 64.32, *p* < .001) (Table 3).

## DISCUSSION

In this study, we defined pain as the stimulating factor and pain as the physiological mode of adaptation using Roy's adaptation model. This study's result was similar to Farsi and Azarmi's study [19] on an OA adaptation program for lower-half amputee veterans. The physiological mode is a physical response that comes from interacting with the environment to meet the basic needs of oxygenation, nutrition, excretion, activity, rest, and protection, and is suitable for evaluating pain by the physical response to pain, which is the focal stimulus of older women with OA.

In this study, perceived health status and depression were set as the self-concept mode for older women with OA. Since Roy’s adaptation model defines self-concept mode as self-perception and subjective thoughts about one’s body [5], it is considered appropriate to adapt perceived health status and depression to the self-concept mode in this study. This study's result was similar to Tsai's study [22], who tested the chronic pain program in older adults with arthritis based on Roy’s adaptation model, and Woo's study [24], who developed a sexual health education program for proctectomy and set depression as the self-concept mode.

This study's result was different from Farsi and Azarmi's study [20], Afrasiabifar et al.'s study [21], and Tsai's study [22], which applied Roy’s adaptation model and set body image, body sensation, and one’s thoughts and emotions about the physical self. Based on this, when developing an adaptation program for chronically ill patients based on Roy's adaptation model, it is judged necessary to add self-concepts such as depression, body image, and body function to the self-concept.

Roy defined role function as skillfully performing a role with a suitable action or interaction with others in the environment, focusing on one’s role [5]. Thus, in our study, the role function mode was set as difficulties in daily life because we judged that those difficulties in daily living for female patients with OA would explain the role function of women as related to housework. Indeed, 51.0% of the participants in this study reported that they perform housework without cohabitants; 51% responded that housework burden was “severe,” and 41.2% responded that housework burden was “middle.” More than 90% of the participants experienced housework burden; therefore, categorizing the difficulty of daily living as a functional role mode was appropriate.

In this study, self-interdependence was measured using social support. This study's result was similar to the findings of Bakan and Akyol's study [23], in which patients with heart failure underwent a heart rehabilitation program. Roy’s adaptation model focuses on self-interdependence related to sharing love, respect, and values. Setting social support in the self-interdependence mode in both studies [24] is thus believed to be valid. In this study, the adaptive responses of older women with OA were set as quality of life. Quality of life was also set as an adaptive response in the study by Afrasiabifar et al. [21], who applied an educational program to patients undergoing hemodialysis. Older women with OA must adapt to their health conditions due to chronic illnesses. Thus, including quality of life in the adaptive responses is valid.

In this study, pain measured in physical and physiological modes was statistically significantly lower in the experimental group than in the control group after the program. These results are consistent with the studies of Lee et al. [7] and Choi and Yoo [25], who reported that the pain in the experimental group was significantly lower than in the control group after a 6-week self-help program for patients with OA. This result was because the program, consisting of exercise, education, and self-help activities, was provided in previous studies, and this study alleviated pain. This result means that the program consists of exercise and education provided in previous studies, and this study alleviated the subjects' pain. In particular, this study thinks that this is because the 12-week exercise program promoted the automatic and unconscious mental and physical response, which is a regulatory mechanism, as physical function improved. The education promoted cognitive information processing, learning, judgment, and emotion coping mechanisms, thereby positively changing the response to pain or pain management.

Among self-concept modes, depression decreased in the experimental group. Depression did not change in the control group; however, there was a significant difference between the two groups. The fact that depression in the experimental group was very low after 12 weeks of the program may have been due to the program’s recreational activities.

In this study, the experimental group's difficulty of daily living showed a statistically significant decrease from 31.12 points pretest to 16.46 points after 6 weeks, and 12.04 points after 12 weeks, showing that the difficulty of daily living decreased for older women with OA. These results were similar to those reported by Kim and Hyun's study [26]. In this study, the depression score decreased after 12 weeks because the program included recreational activities such as hugging partners before the exercise or education program to strengthen cognitive mechanisms, praising partners one by one, clapping, and dancing to a sizzling song during the program. Therefore, we suggest that the adaptation promotion program developed in this study be utilized as a nursing intervention that can reduce depression in patients with OA.

Social support was measured in the self-interdependence mode, and it significantly increased in the experimental group. No relevant studies have been conducted on this measure. The sections of this program that strengthened cognitive mechanisms were thought to increase social support. In this study, social support was measured in the interdependence mode, and the social support of the experimental group increased statistically significantly. It is difficult to make a direct comparison because there is no previous study that measured social support after implementing a program for patients with OA. However, in this study, we think that the increase in social support was the result of the mission of holding hands and praising each other one by one in the adaptation promotion program for older women with OA, and giving one compliment to each grandson, granddaughter, son, daughter, daughter-in-law, and son-in-law at home. As the number of times the subjects participated in the program increased, they said more stories such as, "My grandson or granddaughter said I love you", "My child gave me more allowance", and "My child drove me to the welfare center today". The parts of this program that were implemented as a method to strengthen cognitive mechanisms had the effect of increasing social support.

Adaptive responses in this study were measured based on quality of life. Among adaptive responses, the quality of life of the experimental group tended to increase gradually. This result was similar to the increase in quality of life following self-care programs in studies by Cheon’s study [27], Lee et al.’s study [7], and a tai chi exercise program in Lee and Claire’s study [28]. Quality of life is an adaptive response that occurs after being affected by the four modes of adaptation in Roy's model. This study's OA adaptation program strengthened the regulatory mechanism that improves physical function and the cognitive mechanisms that improve physiological responses, thereby improving the quality of life, which is an adaptive response. It was consistent with the study of Lee et al. [7], Cheon’s study [27], and the study of Lee and Claire [28], which reported that there was a significant difference between the experimental group and the control group after implementing the Tai Chi exercise program. Quality of life is an adaptive response that is affected by the four adaptation modes in Roy's adaptation model, and it is thought that the adaptation enhancement program improved the quality of life, which is an adaptive response, by strengthening the regulatory mechanism that improves physical function and the cognitive mechanism that improves social and psychological responses.

This study has several limitations. Selection biases may have occurred during the selection process, as participants who were able to participate in the program continuously were intentionally assigned to the experimental group. In contrast, those who could not participate were assigned to the control group. Consequently, there may be a lack of homogeneity between the experimental and control groups. Additionally, the study employed a single-blind method; data collectors were not informed about the group assignments of the participants. However, participants in the experimental group were aware of their group assignment, potentially introducing the Hawthorne effect, where participants alter their behavior due to the awareness of being observed. These factors limit the generalizability of the study's findings. Despite these limitations, the study is significant as it demonstrates the potential of applying Roy's adaptation model in the nursing field. It highlights the impact of the OA adaptation program, developed based on Roy's [5] adaptation model, on reducing pain, improving difficulties in daily living, enhancing subjective health status, alleviating depression, and increasing social support among older women with OA. Furthermore, this study is notable for developing and implementing a nursing intervention that integrates physiological concepts, self-concept, and interdependence to improve the adaptation of older women with OA.

## CONCLUSION

This study attempted to confirm whether the OA adaptation program developed based on Roy's adaptation model strengthens the coping mechanisms of older women with OA and promotes adaptation responses. In this study, an integrated approach through exercise and education was attempted to improve the physiological functions, physical functions, self-concept, role functions, and interdependence functions of older women with OA. The results of this study showed that this program strengthened the regulatory mechanisms that improve physical functions and the cognitive mechanisms that improve social and psychological responses, thereby improving the quality of life, which is an adaptation response. In other words, the OA adaptation program based on Roy's adaptation model reduced pain, depression, and difficulties in daily life for women with OA. It improved the quality of life by enhancing perceived health status and social support. The significance of this study is that it proved that Roy's adaptation model can be used as an explanatory framework to improve the quality of life of older women with OA. Therefore, it is suggested that future studies confirm whether the adaptation program developed based on Roy's adaptation model is effective in promoting adaptation in patients with chronic diseases other than OA.

## Figures and Tables

**Table 1.** Homogeneity Test of Characteristics and Independent Variables between Experimental and Control Group (N = 51)

Characteristics		Total	Exp. (n = 26)	Cont. (n = 25)	χ^2^ or t (*p*)
Age (year)^†^	65∼74	16 (31.4)	8 (30.8)	8 (32.0)	1.80 (.459)
	75∼84	30 (58.8)	14 (53.8)	16 (64.0)	
	≥ 85	5 (9.8)	4 (15.4)	1 (4.0)	
		77.78 ± 6.46	78.12 ± 6.87	77.44 ± 6.13	0.37 (.713)
Spouse	Yes	20 (39.2)	10 (38.5)	10 (40.0)	0.01 (.910)
	No	31 (60.8)	16 (61.5)	15 (60.0)	
Educational level	None	18 (35.3)	8 (30.8)	10 (40.0)	1.02 (.600)
	Elementary school	22 (43.1)	11 (42.3)	11 (44.0)	
	≥ Middle school	11 (21.6)	7 (26.9)	4 (16.0)	
Number of children	≤ 3	14 (27.5)	8 (30.8)	6 (24.0)	0.29 (.588)
	≥ 4	37 (72.5)	18 (69.2)	19 (76.0)	
Type of residence	Living with others	25 (49.0)	12 (46.2)	13 (52.0)	0.17 (.676)
	Living alone	26 (51.0)	14 (53.8)	12 (48.0)	
Duration of OA (year)	1∼10	39 (76.5)	17 (65.4)	22 (88.0)	3.62 (.057)
	11∼20	12 (23.5)	9 (34.6)	3 (12.0)	
		8.59 ± 5.83	10.62 ± 5.65	6.48 ± 5.35	2.68 (.010)
Surgery for OA	Yes	16 (31.4)	8 (30.8)	8 (32.0)	0.01 (.925)
	No	35 (68.6)	18 (69.2)	17 (68.0)	
Medical insurance	Employee’s health insurance	36 (70.6)	16 (61.5)	20 (80.0)	2.09 (.148)
	National health insurance	15 (29.4)	10 (38.5)	5 (20.0)	
Functional status	Middle	32 (62.7)	17 (65.4)	15 (60.0)	0.16 (.691)
	Lower	19 (37.3)	9 (34.6)	10 (40.0)	
Burden of homework^†^	Lower	4 (7.8)	1 (3.8)	3 (12.0)	1.36 (.536)
	Middle	21 (41.2)	12 (46.2)	9 (36.0)	
	Severe	26 (51.0)	13 (50.0)	13 (52.0)	
BMI	18.5~22.9	17 (33.3)	10 (38.5)	7 (28.0)	1.91 (.384)
	23.0~24.9	15 (29.4)	8 (30.8)	7 (28.0)	
	≥ 25.0	19 (37.3)	8 (30.8)	11 (44.0)	

Values are presented as the mean ± standard deviation or n (%). Exp. = Experimental group; Cont. = Control group; OA = Osteoarthritis; BMI = Body mass index.

† Fisher’s exact test.

**Table 2.** Homogeneity Test of Dependent Variables between Experimental and Control Group (N = 51)

Variables	Total	Exp. (n = 26)	Cont. (n = 25)	t (*p*)
Pain	6.63 ± 1.44	6.85 ± 1.43	6.40 ± 1.44	1.11 (.274)
Difficulty of daily living	28.25 ± 12.10	31.12 ± 9.08	25.28 ± 14.17	1.74 (.089)
Depression	4.53 ± 3.18	4.58 ± 3.50	4.48 ± 2.87	0.11 (.915)
Social support	20.67 ± 3.15	21.50 ± 3.33	19.80 ± 2.77	1.98 (.053)
Quality of life	94.82 ± 10.57	94.88 ± 10.77	94.76 ± 10.58	0.04 (.967)

Values are presented as the mean ± standard deviation. Cont. = Control group; Exp. = Experimental group.

**Table 3.** Effects of the Adaptation Promotion Program for Older Women with Osteoarthritis (N = 51)

Variables	Groups	Pretest	Posttest 1 (after 6 weeks)	Posttest 2 (after 12 weeks)	Effects of program		Difference t (*p*)	
					Sources	F (*p*)	Pretest vs. posttest 1	Posttest 1 vs. posttest 2
Pain	Exp.	6.85 ± 1.43	5.23 ± 0.86	4.46 ± 1.07	Group	12.10 (< .001)	5.70	3.67
	Cont.	6.40 ± 1.44	6.44 ± 1.16	6.44 ± 0.96	Time	5.93 (.007)	(< .001)	(< .001)
					Group×Time	23.65 (< .001)		
Difficulty of daily living	Exp.	31.12 ± 9.08	16.46 ± 5.98	12.04 ± 4.44	Group	3.88 (.055)	11.63	5.21
	Cont.	25.28 ± 14.17	25.16 ± 11.52	21.92 ± 9.44	Time	14.54 (< .001)	(< .001)	(< .001)
					Group×Time	39.57 (< .001)		
Depression	Exp.	4.58 ± 3.50	1.65 ± 1.38	0.81 ± 0.63	Group	7.24 (.010)	5.30	3.28
	Cont.	4.48 ± 2.87	4.20 ± 2.24	3.16 ± 1.95	Time	11.13 (.001)	(< .001)	(.003)
					Group×Time	9.17 (.002)		
Social support	Exp.	21.50 ± 3.33	24.73 ± 1.61	26.42 ± 0.76	Group	46.07 (< .001)	－6.76	－5.03
	Cont.	19.80 ± 2.77	19.24 ± 2.89	19.76 ± 2.33	Time	22.83 (< .001)	(< .001)	(< .001)
					Group×Time	45.22 (< .001)		
Quality of life	Exp.	94.88 ± 10.77	106.15 ± 8.62	115.73 ± 4.13	Group	29.04 (< .001)	－10.95	－7.11
	Cont.	94.76 ± 10.58	91.28 ± 8.23	93.12 ± 4.59	Time	19.08 (< .001)	(< .001)	(< .001)
					Group×Time	64.32 (< .001)		

Values are presented as the mean ± standard deviation. Cont. = Control group; Exp. = Experimental group.

## Data Availability

The data that support the findings of this study are available from the corresponding author upon reasonable request.
